# Design of a Kitchen-Monitoring and Decision-Making System to Support AAL Applications

**DOI:** 10.3390/s21134449

**Published:** 2021-06-29

**Authors:** Nikola Žarić, Milutin Radonjić, Nikola Pavlićević, Sanja Paunović Žarić

**Affiliations:** 1 Faculty of Electrical Engineering, University of Montenegro, 81000 Podgorica, Montenegro; mico@ucg.ac.me; 2 Technical Support Office, ZTE Corporation, 81000 Podgorica, Montenegro; nikola.pavlicevic2@zte.com.cn; 3 Faculty of Architecture, University of Montenegro, 81000 Podgorica, Montenegro; sanja.paunovic@ucg.ac.me

**Keywords:** ambient assisted living, decision-making system, cooking process monitoring, ultrasound sensor, temperature and humidity

## Abstract

Numerous researchers are working on Ambient Assisted Living systems to enable more comfortable and safer living for senior people in their homes. Due to modern lifestyles and an aging population, this has become a very important issue. According to the available literature, it is obvious that the kitchen is one of the most important and most dangerous rooms in the home. However, there is still evident lack of monitoring systems suitable for specific kitchen activities. In this paper, we propose a monitoring system capable of identifying activities related to the cooking process, and a decision-making system capable of identifying some unwanted and possibly critical conditions. The proposed systems are designed to satisfy the requirements of the modern Ambient Assisted Living systems dedicated to older adults. The proposed monitoring system consists of ultrasound, temperature, and humidity sensors. The acquired results from these sensors are the inputs for the decision-making system, which generate warnings or alarms intended for the senior users and/or formal or informal caregivers. This system is designed to improve home safety related to kitchen activities, as well as to provide information about the lifestyle and daily activities of senior users. Experimental validation of the proposed system confirms its functionality and accurate design approach.

## 1. Introduction

The aging human population is a well-known problem in developed countries, and things are becoming more complicated. Many circumstances, such as healthier lifestyles, improved education, effective birth control, and the growth of the global population have caused the share of the people aged 65 or over to increase. Some estimations show that this trend will continue. There are some projections by the European Commission that show the rising of the EU population from 495.4 million in 2008 to 520.7 million in 2035, and thereafter gradually falling to 505.7 million by 2060. “The share of people aged 65 years or over in the total population is projected to increase from 17.1% to 30.0% and the number is projected to rise from 84.6 million in 2008 to 151.5 million in 2060. Similarly, the number of people aged 80 years or over is projected to almost triple from 21.8 million in 2008 to 61.4 million in 2060” [1]. There is a very similar trend in other developed countries.

In recent years, the number of people affected by various diseases has been significantly increasing. Elderly people very often suffer some physical or cognitive impairment, which becomes worse as they grow older. Over the years, the power of concentration, timely reaction, memory, rational reasoning, and many other abilities that are important for routine activity are slowly lost. Consequently, the ability to live independently, in the sense of performing daily activities, decreases with age.

The World Health Organization reported in 2010 that about 35.6 million people lived with dementia worldwide [2]. Approximately 7.7 million new cases occur each year, which means that the estimated number of the elderly people with dementia will be 65.7 million in 2030, and 115.4 million in 2050. According to [3], one in five people older than 65 suffer from Mild Cognitive Impairment (MCI), which means a noticeable decline in cognitive ability (such as memory, decision-making, problem-solving, and comprehension) that does not prevent the carrying out of daily activities. A very similar situation arises with other diseases. Moreover, older people usually require intensive health care and more specialized services, which means that health system costs are set to rise significantly.

On the other hand, it is human nature to be attached to one’s accustomed environment. Thus, older people find it difficult to decide to go into nursing homes or similar institutions that care for the elderly, instead preferring to stay independently in the familiar environment of their own home. The financial aspect of this decision is not negligible either, as staying in special institutions requires additional costs. Studies have shown that 89% of older adults would prefer to stay in the comfort of their own homes [4]. However, there are not enough trained staff to work with elderly people, especially those with disease. 

Therefore, several strategies have been developed to promote the independent life of older people in their own homes for as long as possible [5]. In a United Nations report from 2013, it was stated that older people live alone or only with a partner in about 40% of the total older population [6]. Since other family members often do not live nearby or even in the same city or same country, senior people are required to take care of themselves almost without any external help. Additionally, there is frequently a lack of formal caregiver services, or senior people cannot afford it due to limited financial resources. Independent living becomes even harder if there are physical or health constraints. Therefore, especially in the recent years, a lot of effort and resources have been invested in the development of systems for helping elderly people. Such systems usually incorporate sensors, actuators, controllers, information and communications technologies (ICT), and other assistive technologies [7]. A common name for such a system is an Ambient Assisted Living (AAL) system [8].

With the development of portable devices such as mobile phones, tablets, smart watches, etc., the possibility of applying modern technologies is expanding, and the development of home assistance systems is improving. Although older people are often not ready to accept new technologies and learn how to use AAL systems, these portable devices have become an integral part of everyday life and can be used by almost everyone, without dedicated training. Since portable devices are very often equipped with many sensors suitable for AAL application, their integration into a system is a logical choice [9]. Moreover, it is expected that control of almost all home appliances and devices through a mobile phone application will become standard very soon. 

In recent years, smart-home technology has developed rapidly and has been increasingly applied. This technology primarily aims to improve the quality of life of all categories of residents in urban areas. Smart homes are typically equipped with sensors/actuators, communication infrastructure, and information management systems, enabling some degree of either automation or remote control. Most popular appliances suitable for smart-home paradigms are refrigerators, ovens, cookers, washing machines, air-conditioners, garage or courtyard doors, security systems, entertainment systems, lighting systems, and so on. With the advent of smartphones, managing a smart home has become very simple. As well as the ability for various processes to be started (such as laundry or air-conditioners, for example) when the user is not at home, it is also possible to achieve significant savings from large electricity consumers in periods of discounted electricity prices. Furthermore, smart-home systems can log operations so that user habits and resource use can be analyzed for the better optimization [10]. 

Various sensor technologies are currently available for smart-home applications. They observe a person’s interaction with home appliances and the other objects mentioned above, as well as movement around the home. Based on the collected information, a projection of user habits is performed [11]. Such information can be conveniently used to improve the performance of AAL systems.

Modern technology is used to help solve the problems of chronic disease, which often affect the elderly [12]. Furthermore, systems are being developed to improve the quality and ensure the safety of the older people living independently in their homes. As a result of work on multinational projects, systems and solutions that make a significant contribution in this regard have been created, and are based on the application of modern technology [7,13,14,15,16]. 

The kitchen is the room in the house where many accidents occur, often causing injuries, especially in the case of the elderly and people with dementia. Therefore, monitoring the processes that take place in and around the kitchen is a very important aspect of a system whose task it is to facilitate and make the independence of elderly people in their homes safer [17]. One of the most important segments in the daily routine at home is food preparation. Proper nutrition is of the great importance for human health, and activities related to nutrition can also serve as an indicator that a person can independently perform basic activities in the intended manner. Thus, monitoring the use of kitchen appliances is important from at least two aspects. In addition to checking that the person has eaten every meal, this monitoring can provide additional information on unwanted and potentially dangerous events.

Studies have shown that the cooker is the leading cause of the fire in home. Cooking accidents are responsible for about 31% of home fire, where the main factor is an unattended cooking process [18]. Analyzing the behavior of persons suffering from MCI, many risky situations and other difficulties handling the cooker have been identified [18,19]. Therefore, it is necessary to improve kitchen-monitoring and control, not just for the elderly and people with disabilities, but for the population in general [20].

Let us first review solutions that can be found on the commercial market. Modern commercial smart-home systems include various sensor solutions such as monitoring kitchen cabinets, kitchen utensil use, user movements, and use of different appliances. Simple kitchen devices such as coffee machines, water heaters, and some more complex devices such as refrigerators, washing machines, microwaves, and ovens are becoming smarter, providing certain information about user interaction with these devices. Regarding the hotplate, which is in the focus of our research, there are only a few smart features provided in commercial solutions, such as remote on/off functions and the setting of appropriate plate temperature [21,22,23]. Additionally, solutions with glass ceramics have an anti-spill safety function and a system that automatically reduces the temperature when the plate is overheated. However, these features are performed automatically and do not provide additional information that can be used by the decision-making system or some other module of the complex AAL system. Moreover, commercial solutions cannot detect user interaction with the cooking process. Thus, the researcher community has made significant efforts to develop a more dedicated cooking-monitoring process [17,18,19,20,24,25,26,27,28].

There are several proposed approaches and solutions related to the improvement of safety in the kitchen. Some are focused on smoke or gas detection to prevent fire and/or intoxication, as well as temperature, humidity, and ultrasonic distance detection to reduce burn risk [24]. In addition, some proposals include sound sensors for water monitoring, timers for medication dispensers, and even current-flow detection for the monitoring of appliance operating times [25]. 

One of the possible approaches for monitoring the process in the kitchen is the use of thermal cameras [18,26]. In this way, the temperature of objects in the kitchen can be monitored, primarily the stove. These systems usually use thermal images to establish the correlation between burner temperature and presence of a pot over it. Cookware presence detection is proposed in [24,25], but with the use of the ultrasound sensors. These methods commonly include PIR sensors for movement detection along with temperature and pot presence detection. However, all these methods have some drawbacks. Some are not preventive, and consequently do not meet the safety criteria, such as smoke detection, for example. Some others, such as temperature and humidity analysis, are not reliable enough. Finally, a thermal camera cannot be easily used to monitor the entire food preparation process and draw conclusions about possible omissions that may occur to the elderly. Hence, using a thermal camera is good, but it is an expensive and non-comprehensive solution.

With the advent of the Internet of Things (IoT), more and more solutions rely on this technology. The authors in [27] used the Arduino platform for the development of a fire prevention system in the kitchen that relies on several types of sensors. It contains an alarm system that can send messages to relevant services, and a camera that can monitor the kitchen over a mobile phone. However, this solution is primarily intended for kitchens that have gas appliances.

Another piece of hardware architecture in the safe-cooking system that targets the enhancement of the safety of elderly people while cooking is presented in [28]. This system is based on the monitoring and measurement of several parameters during cooking and, according to these parameters, a risk analysis is performed. The paper presents the results of an experimental study that was the platform for the selection of appropriate sensors for the realization of the system. The idea was to react proactively to possible safety risks associated with the cooking process.

Another very important aspect of the AAL system is related to the appropriate analysis of the data received from sensors through the decision-making process. Specifically, pure sensor data are sufficient only for triggering alarms for critical situations such as fire detection [11,14,24,25,26,27,28,29,30], smoke [11,14,24,26,27,28,29], and gas leakage [11,24,25,29]. To obtain information about daily user activity, more complex analysis and decision-making systems are required. Regarding the recognition of general AAL-related activities, there are many studies that include various sensing devices. Modern methods of the machine-learning have been used recently to recognize certain human activities as quickly and as reliably as possible, and to take necessary actions within the system itself. It is especially important to recognize more complex activities that can only be detected by the joint use of different, and often very diverse, sensors [29]. In addition, advanced and intelligent mining techniques can be used to detect irregularities within a system or to detect the malfunction of individual components [31]. When it comes to activities in the kitchen, there are studies related to the recognition of activity such as eating [11,14,17,29], drinking [11,17,29], taking medication [14,17,29], and similar. Additionally, there are studies related to the analysis of the quality of prepared food, but not in the context of elderly people and their needs and habits [32].

Decision-making systems are responsible for thorough analysis of sensor data and triggering appropriate alarms and notifications. Furthermore, decision-making systems can provide information for the rest of a typical AAL system. In that case, it is possible to track overall user behavior regarding daily activity.

Despite an intensive review of the literature, we found a lack of systems that adequately and comprehensively monitor the processes related to the kitchen. The food preparation process is particularly poorly analyzed and evaluated, especially possible problems related to the handling of stoves by elderly people. This has motivated us to consider that problem, and to contribute to the design and implementation of a system that could monitor processes related to food preparation and to warn or alert in the case of suspicious or dangerous situations. To better understand kitchen activities and possible unwanted situations, we have performed a detailed analysis of the cooking process and segmentation in different scenarios. That leads to the design of the decision-making algorithm, which will provide relevant information to the rest of the AAL system. Moreover, the system that we propose in this paper aims to contribute to the enhancement of quality of life for elderly people. Specifically, the novel decision-making system is capable of observing the cooking process from beginning to end, and leading the user successfully toward finishing. 

The rest of the paper is organized as follows. Section 2 addresses the position of kitchen elements from an architectural point of view, as well as detailed analyses of cooking process use cases. A decision-making system is proposed in Section 3. The implementation of the monitoring system is considered in Section 4. The experimental results are presented and discussed in Section 5, while concluding remarks are given in Section 6.

## 2. Analysis of the Kitchen Setup and Cooking Process

### 2.1. Analysis of the Kitchen Environment

Activities in the kitchen, such as beverage and meal preparation, are mandatory daily activities for each individual who lives independently at home. There are several studies that analyze daily user activity in residential spaces. It has been shown that on average 30% of activities in the home are subordinated to the food preparation, and that 360 different actions are performed in the kitchen during the day [33]. Elderly people often have physical limitations, alongside the cognitive ones, so reducing movement and preparing the conditions for efficient use of the kitchen workspace is very important. People with limited physical abilities are usually unable to move heavy kitchen items from one place to another, so the continuity of the working space seems to be very important in this process. 

To provide a safe and useful kitchen space for senior people, it is sometimes desirable to make certain adjustments. Having in mind that the average expiration period of a kitchen is about 20 years, it is easy to justify renovation. Additionally, the kitchen has been identified as the most desirable space for renovation, both aesthetically and technically. According to [33], 34% of people prefer to renovate their kitchen before the other areas in the home. To the best of our knowledge, current research related to AAL did not take into account the dimensional analysis and design of the existing kitchens, as well as the possibility of considering the whole process of the food preparation and sensor position. The adaptation of the environment to user needs is always determined by space limitations. New product lines that support mobility, interactivity, simplicity, accessibility, safety, and comfortable use of the senior people’s environment, with the possibility of monitoring, are desirable and necessary not only in private apartments, but also in specialized institutions. 

Most kitchen activities are performed in the area in between sink/dishwasher (washing), fridge (storage), and cooking hob, which makes a “working triangle” [34]. It is almost inevitable that a cooker will be used in this process. 

The most important aspects to be considered during kitchen design intended for elderly people are recognized as:

− spatial analysis of the existing kitchen, such as dimensions;
− logical configuration of functional zones, correct working triangle, intuitive and simple layout regardless of user cognitive capabilities;
− distance between functional zones, which is essential for reducing physical effort. The design should be efficient and comfortable, with a minimum of fatigue [35].

One of the most important aspects is continuity of the working surface. Kitchens that have an interruption in the continuity of the food preparation process are especially unfavorable for people who are weak or who use wheelchairs, because moving hot and heavy objects above a countertop becomes almost impossible [36]. Another important issue is the flexibility of use. Design should take into account, as much as possible, a wide range of individual preferences and abilities, mostly the ability to adapt an existing space to the people who need help or those in a wheelchair [35]. 

**Figure 1:**
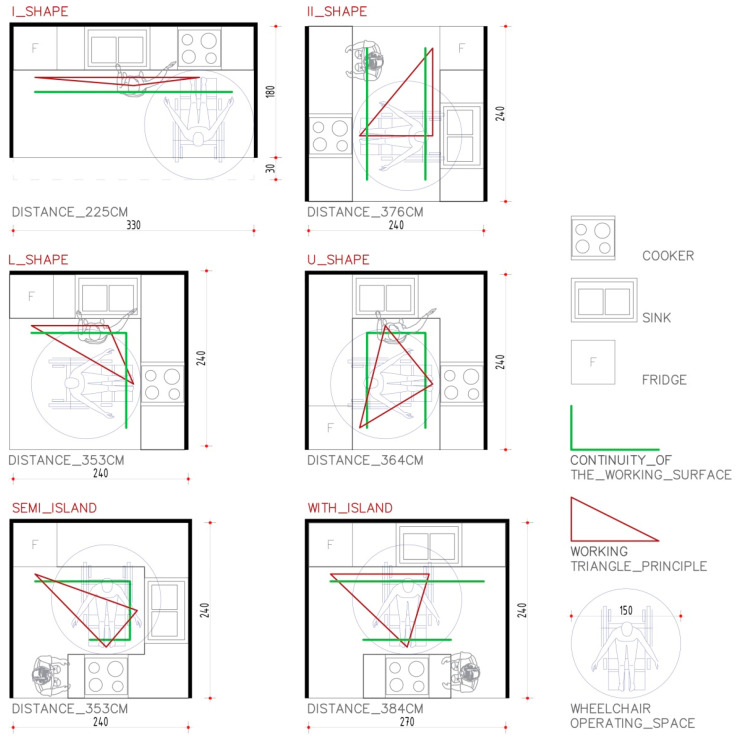
Six characteristic types of kitchen related to the distance of countertop continuity and optimization of 3 main kitchen elements that create a working triangle.

Six typical types of the kitchen are shown in Figure 1. These kitchens are further analyzed in terms of user movement inside the kitchen and use of the cooker as the primary considered element in this research. The II_SHAPE form of kitchen, and kitchens with an island, have discontinuities of countertop and significantly larger distances between kitchen elements. I_SHAPE and L_SHAPE have a logical position of elements over short distances, which can easily support any user regardless of the physical capabilities. These two shapes also provide a good overview of all subsequent elements in the kitchen (front or side view and access, without needing to rotate 180 degrees as in the U-SHAPE, kitchen with semi-island, and kitchen with island). Therefore, the organization of the cooking process becomes easier. In this way, the user is stimulated to do several related actions during the cooking process simultaneously. According to the previous discussion, it was concluded that the I_SHAPE and L_SHAPE of kitchen are the most suitable, both in terms of distance and continuity of countertop. Additionally, the I_SHAPE and L_SHAPE of kitchen are most suitable for people in wheelchairs, since there is enough space for daily food preparation activities.

In accordance with the optimization of kitchen activities related to the three main elements of the kitchen equipment, it is desirable to rethink and optimize the sensor positioning zone. A set of standard sensors for emergency detection, such as gas sensors, smoke detectors, flood detectors, etc., are usually mounted on the ceiling. Seniors do not like to be aware of constant monitoring, so mounting sensors in the user field of view is inconvenient. Although electronics manufacturers strive to produce equipment that is light, durable, and stable to use, it requires constant upgrading in terms of servicing and functionality [37]. This implies that users must be able to implement these new features quickly, by adding certain devices to existing system, not by complete replacement [17]. 

Since AAL systems require the implementation of various monitoring sensors, the six basic types of the kitchen are analyzed in terms of installation efficiency and complexity. The green line of Figure 1 shows the appropriate fields for sensor position. Installation of sensors on the kitchen wall would result with visible and accessible cables. This could disturb the user because these cables and corresponding sensors are in the user field of view. Thus, a better position for sensor placement is in the body of the kitchen cabinets. Behind these elements it is easy to install, hide, and protect required sensors and corresponding cables. Additionally, access to cables and sensors is convenient if some modification or replacement is required. Additionally, the coverage area of the sensors is an important issue that should be taken into consideration. Specifically, if the most important kitchen elements are close to each other, then the number of the sensors can be reduced. Thus, the total cost of AAL hardware components is also reduced. Therefore, one can conclude that the I_SHAPE and L_SHAPE kitchens are better choices from the point of complexity and installation. Kitchens with an island and a semi-island are recognized as inconvenient from the point of sensor installation, due to the longer distances between kitchen elements. Additionally, they are usually equipped with special hoods, without upper kitchen elements above the island. Therefore, positions for the sensors are limited to the ceiling surfaces. 

### 2.2. Analysis of the Cooking Process

Activities in the kitchen, such as beverage and meal preparation, are mandatory parts of daily activity for every individual who lives independently at home. It is almost inevitable that a cooker will be used in this process. Since AAL systems are focused on elderly people and people with some cognitive disabilities, such as dementia, using the cooker might cause various unexpected and possibly dangerous situations. Equally important is the success of the cooking process and possible failures that may affect the mood of elderly people. If the cooking process fails, or possibly even did not start properly, users might become disappointed and nervous, which may cause other side effects, such as consumption of unhealthy food that does not require a complex cooking process, or even refusing to eat. Thus, assisting in food preparation and verification that the cooking process has successfully been completed is as important as the detection of possible unsafe and dangerous situations.

Since the cooking process might be a complex and sometimes time-consuming activity, various scenarios related to plate statuses and the corresponding user activities can be identified. In the following analysis, *Status* will refer to the plate setup (on/off, with/without pot on it), which is detected by the corresponding sensors. For each *Status*, multiple use cases are analyzed. Use cases (in the analysis labeled as *Cases*) are related to user interactions with the cooking process, status of the cooking process (boiling, evaporation), elapsed time, and previous statuses of the plate itself. Detailed analysis is presented in the following.

***Status 1:***  Plate is turned off and there is no object on it.

**Case 1.1**: There are no temperature changes or user activities detected. 

This is considered to be the regular case and no reactions of the system are required. 

**Case 1.2**: Higher temperature than usual is detected.

This indicates that the cooking process has recently finished, and that plate is still hot.

***Status 2:***  The plate is turned off but there is an object on it.

This condition on the hotplate is mostly considered to be regular, but might indicate several unwanted situations that possibly require reaction of the system. 

**Case 2.1**: A pot is detected on the plate but there are no further user activities. 

This might indicate that the user forgot to turn on the plate. This is identified as a fairly common situation, possibly caused by interruption to the cooking process due to some other activity. For example, the user might receive a phone call, needed to go to the toilet, care for a pet or some other stimulus. To avoid user anxiety when realizing that the cooking process did not start as expected, the system should notify the user that they might have forgotten to turn on the plate. If there is no user reaction to this notification, the system will not take any further action. However, this information will be saved within the system for further analysis of the user activities and personal habits.

**Case 2.2**: A pot remains for a defined specific time on the plate after completion of the cooking process. 

This is considered to be regular situation, and no reaction or notification to the system is required.

**Case 2.3**: A pot remains on the hotplate for longer than a defined specific time.

In this case, the system should ask the user about the status of the pot. Specifically, it might indicate that the user forgot to eat the prepared food. Additionally, if the food remains on the pot for too long, it might become unusable or could produce an unwanted odor. Information about this event is saved within the system for further analysis. If this happens frequently, it might indicate a loss of cognitive capabilities, or irregular food consumption, or even inappropriate hygiene in the kitchen.

***Status 3:***  A plate is turned on but there is no object on it.

This is possibly a very dangerous situation that may happen at the begging or at the end of the cooking process. 

**Case 3.1**: There is no user reaction within a specific time. Two possibilities could be identified:

- the user might have forgotten to put the pot on the plate (disrupted by something, as mentioned in Case 2.1).
- the user put the pot on one plate while the other is turned on. This is quite frequent, according to our experience.

**Case 3.2**: The user forgot to turn off the plate after completion of the cooking process (which does not happen only to elderly people). 

In any of these cases, the system should wait a certain time for the user reaction. If there is no user reaction, the system should warn the user to put the pot on the active plate or to turn off the plate. If there is still no user reaction for a certain time, the system should perform an appropriate emergency action. This emergency action could be performed according to the system implementation and configuration. If there is an option to turn off the plate, this would be the most appropriate action. If the system is not equipped with this option, an informal caregiver or emergency service should be notified. In either case, the term “emergency action” will be used in the rest of the paper to denote this activity.

***Status 4:***  The plate is turned on and there is an object on it.

This is considered to be an ongoing cooking process. As already mentioned, cooking is a complex and time-consuming process that requires user interaction. Therefore, various cases might be identified.

**Case 4.1**: There is a constant distance, and no significant variation in temperature or humidity is detected. Additionally, user interaction with the cooking process could be detected (movement of a hand, use of mixing spoon, lifting a lid, etc.). This indicates a regular cooking process that should be further monitored. 

**Case 4.2**: There is constant distance detected, but significant increase of humidity occurs. This indicates that liquid is probably going to boil. 

**Case 4.3**: Constant increase or decrease of the content level (distance) in the pot is detected. Increase of the content level (usually followed by increased humidity) might indicate possible boiling, while constant decrease might indicate evaporation.

**Case 4.4**: Constant distance is detected for a long period of time (or approximately constant if the pot is without a lid). This indicates that the user did not interact with the cooking process during that time, which means that they might have forgotten about it. 

Case 4.1 is considered to be regular, and it does not require any system reaction, while others are identified as possibly critical and require user notification. The main role of these notifications is to lead the user toward the successful completion of the cooking process and to avoid undesirable and possibly dangerous situations, such as long-lasting boiling and evaporation. For example, Case 4.4 may cause overcooking of the food, while in the Case 4.3 food might become burnt or even charred. In both cases, food is at least un-tasty and more often inedible, which leads to user disappointment. A more dangerous situation is boiling, since it may cause user burns. If the failure of the cooking process repeats frequently, the user might lose self-confidence, tend to avoid preparing food, and consequently have irregular meals.

## 3. Automated Decision-Making and Analysis System

AAL systems are designed to continuously monitor user activities, prevent or detect possibly dangerous situations, and perform different analyses and draw conclusions and suggestion aimed to improve user quality of life. Therefore, obtaining information about frequency, duration, and success/failure of the daily cooking process become very important for analysis of the user daily activity. 

To automatically analyze data from the monitoring system and to provide valuable information to the rest of the AAL system, we propose a decision-making system. This is designed to identify all cases addressed in the previous section. Focus is placed on the notification system that should warn the user in a way that stimulates and guides the successful completion of the cooking process and minimizes the risk of any possible dangerous conditions arising. For the sake of simplicity, the decision system is designed for each plate individually, since the sensing system we designed (presented in the next section) consists of individual sensors for each plate. 

### 3.1. Input Parameter Analysis

**Figure 2:**
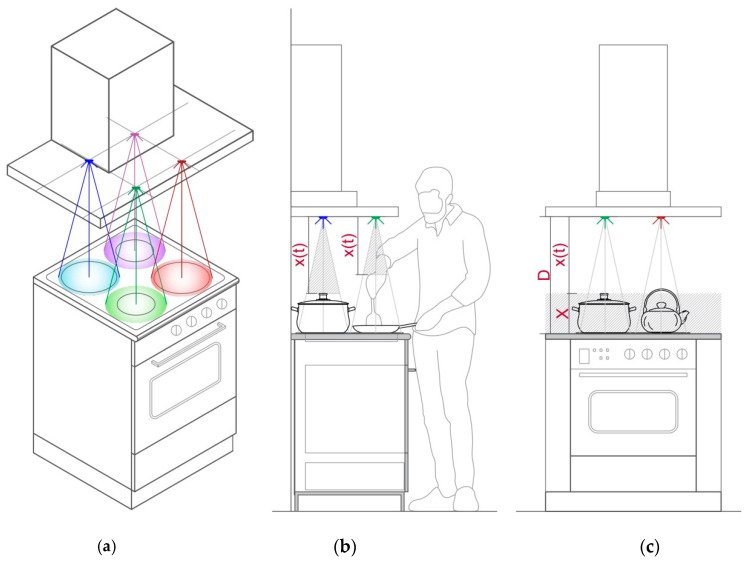
Illustration of the sensing system and corresponding distance measurement parameters; (**a**) axonometric view with the position of sensors, as well as relation between cooking area and hood; (**b**) side view; (**c**) front view.

The decision-making system operates depending on the number of input parameters: distance, plate status, temperature, humidity, time, and variations of some of these parameters. Input parameters are obtained from the corresponding sensors, or calculated based on acquired information. The position of some sensors is illustrated in Figure 2a, while the experimental setup is presented in the next section. Detailed analysis of the input parameters and their values is presented as follows.

#### 3.1.1. Distance Parameter—*d*

To determine the presence or absence of an object on the plate, we attached an ultrasound sensor mounted on the hood, as shown in Figure 2. The value of the ultrasound sensor reading represents the distance parameter, labeled as *x*(*t*), measured in cm. The distance between the ultrasound sensor and the empty plate surface is constant and denoted as *D*. Since there are number of cooking utensils that differ in height (from a few centimeters up to 20 or 30 cm), we decided to determine the zone where we assume the utensil might be identified, as illustrated in Figure 2c. This zone is denoted as *X*. Based on the measured distance, several possible inputs for the decision-making system are identified and summarized in Table 1.

#### 3.1.2. Plate-State Parameter—*p*

Information about whether the cooker, or any individual plate, is switched on or off can be obtained using a Hall sensor, which can detect current flow. If the power consumption of each burner is known, and if it is different for each burner’s circuit, it is possible to determine exactly which circuits are active [20]. A design of a circuit for contactless and precise AC-current sensing using a Hall sensor is given in [38]. We will consider this information as the plate state. The plate state is denoted as *p*, and could have two values:

*p* = 0—plate turned off,
*p* = 1—plate turned on.

#### 3.1.3. Alarm Parameter—*a*

This parameter is responsible for triggering notifications about possibly dangerous conditions. This is a complex parameter that depends on the plate state, distance, and time elapsed from a certain event. As discussed in Section 2, there are several cases when the system should notify the user about the status of the cooking process based on the time elapsed. Thus, this will be analyzed in more detail (Table 2). 

In the Case 2.1, the plate is turned off and an object is detected on it, but there are no further user actions within the predefined time period denoted as AT_1_. This will trigger a user notification to check the status of the pot and the plate. Since this is non-critical condition, no further action is required. However, information about the triggering of a notification and the corresponding timestamp will be the system output and stored within the AAL system for further analysis, as described in Case 2.3. 

If Status 3 occurs (regardless of the case) and lasts more than the predefined alarm threshold AT_2_, the system should notify the user that the plate is turned on. Since this is possibly a dangerous condition, the alarm threshold AT_2_ should be quite short. 

Another case identified based on the time elapsed from certain state change is Case 4.4. Specifically, if the user does not interact with the ongoing cooking process for a longer than predefined alarm threshold AT_3_, the system should notify the user to pay attention to the cooking process. It is quite difficult to precisely determine this threshold due to numerous possible food preparation time requirements, but some period between 20 and 30 min is quite reasonable.

Finally, if there is no prompt user reaction to the notifications in any of the previous cases (except Case 2.1) the system should start new timer *t_c_*, which is the timer for critical conditions. If there is no user reaction to the notification before the critical alarm threshold AT_c_ elapsed, the system should perform emergency action. Information about starting the critical timer, together with the corresponding notification, will be considered to be the output of the proposed system toward an AAL system. It will be used to observe the frequency of repetition of such an event, which might provide very useful information about the cognitive capabilities of the user. 

Selecting the appropriate alarm threshold time is not an easy task, and should require specialists from different fields, which is beyond the scope of this paper. We will propose some alarm threshold times for experimental validation of the system, but their variation will not disrupt the functionality of the decision-making system. Different alarm threshold times could make the decision-making system to faster or slower respond to certain user activities. For experimental purposes, we selected the following alarm threshold times:

- *AT*_1_—5 min—the user might have left the pot on the plate for a certain time while preparing an ingredient for cooking;
- *AT*_2_—3 min—this is possibly a critical condition, and a shorter time period is selected;
- *AT*_3_—20 min—it is a reasonable time for a regular cooking process without the need for user interaction;
- *AT_c_*—2 min—an emergency action period is shortened due to security reasons.

#### 3.1.4. Warning Parameter—*w*

This is quite a complex parameter, but the one responsible for the success of an already started cooking process. Specifically, by introducing this parameter to the decision-making system, we can identify or prevent boiling and evaporation as two events that may cause the failure of the cooking process. Additionally, if there is no timely user reaction, these events might even become dangerous if they last too long. For the identifications of these boiling and evaporation events, distance and humidity parameters are considered as well as a warning timer denoted as *t_w_* (Table 3). 

The boiling condition may occur in two scenarios. The first is when there is a lid on the pot. In that case, the constant distance is detected, but humidity (*h*(*t*)) significantly increases over the considered time period. The second scenario is when there is no lid on the pot. In that case, a constant increase (distance decrease) of the content level is detected, followed by an increase in humidity. In any of these scenarios, the time period prior user notification should be quite short to avoid undesired boiling. Let us name this boiling warning time and denote it as *WT_b_*. The boiling warning time should not be longer than 1 min.

Regarding evaporations there are also two possible scenarios. If there is no lid on the pot, evaporation might be identified as a constant level decreasing (distance increasing). Detection of an evaporation event when there is a lid on the pot is much more difficult with the proposed sensors. In this case, distance is constant, while evaporation might be detected by decreased humidity and somewhat increased temperature, which probably means that the food has already evaporated. Therefore, for precise identification of this case of an evaporation event, some additional sensors, such as that for the detection of a mixture of gases, should be introduced. Since evaporation is a much slower process than boiling, evaporation warning time (*WT_e_*) should be about 3 min.

If there is no user reaction within the predefined intervals of *WT_b_* or *WT_e_*, the system should run a critical event, and start critical timer *t_c_*, as previously described.

### 3.2. A Decision-Making Finite-State Machine

During the initial consideration of the system design, several possibilities are taken into account. After extensive analysis of various approaches, we decided to design a decision-making system using a Moore finite-state machine. It is simpler and faster than other techniques. At the same time, it provides sufficient functionality, and it is convenient for hardware implementation. A decision-making system designed as a Moore finite-state machine is presented in Table 4. States are selected to represent identified use cases, while outputs are generated to trigger notifications as well as to indicate the success of the cooking process. 

States that are identified to implement this machine are:

Q0— initial state—wait to start the cooking process—Case 1.1;
Q1— pot is detected on the plate that is turned off—Case 2.1;
Q2— notify the user that the object is on the plate that is turned off—Case 2.2
  (Please note that Case 2.3 is not among the considered states, since it represents the segment of the analysis module of an AAL system);
Q3— plate is turned on—Case 3.1;
Q4— plate is turned on, but no object is on it—Case 3.1;
Q5— user notification—reaction to Case 3.1;
Q6— reaction to Case 3.1 that is part of emergency module of an AAL system;
Q7— cooking process started—Case 4.1;
Q8— boiling or evaporation started—Case 4.2 or Case 4.3;
Q9— user notification—reaction to Case 4.2 or Case 4.3;
Q10— reaction to Case 4.2 or Case 4.3 that is part of the emergency module of an AAL system;
Q11— no user interaction with the cooking process—Case 4.4;
Q12— user notification—reaction to Case 4.4;
Q13— reaction to Case 4.4 that is part of the emergency module of an AAL system;
Q14— cooking process is finished—Case 1.2.

Proposed decision-making system generates the following outputs:

O_0_ = 000—cooking process did not start;
O_1_ = 001—for State Q2—notify the user that the pot is on the plate that is turned off;
O_2_ = 010—for State Q5—notify the user that the plate is turned on, but there is no pot on it;
O_3_ = 011—for State Q9—notify the user about the boiling or evaporation process;
O_4_ = 100—for State Q12—notify the user that there is no interaction with cooking process;
O_5_ = 101—emergency action;
O_6_ = 110—ongoing cooking process, continue monitoring;
O_7_ = 111—cooking process finished.

## 4. Implementation of the Experimental Setup

The first step toward the implementation of the system, whose concept has been detailed in the previous sections of this paper, is to determine whether the anticipated sensors can provide useful and accurate information. In this work, the goal of the experiment is to receive the necessary data from sensors, which are then analyzed, to identify the appropriate inputs for the decision-making system. For that purpose, we have designed a hardware setup, which consists of two parts—the first is the circuitry located in the kitchen, in the hotplate zone, and the second part is the remote side. 

The hotplate zone setup consists of the four ultrasound sensors, two pairs of temperature and humidity sensors, a microcontroller, and a Wi-Fi module. On the remote side, there is a microcontroller platform connected to a PC. This platform has an integrated Wi-Fi module. The two sides communicate over the Wi-Fi connection. Data obtained from sensors are transmitted to the remote side for further analysis and processing. This facilitates a testing process and allows us to monitor the hotplate zone remotely. 

The hardware used in this experiment is:

- 4 pieces of ultrasound sensor HC-SR04 [39].
- 2 pieces of digital temperature and humidity (DHT) sensor DHT22 [40].
- Wemos D1 R1—development board with ESP8266 microcontroller and Wi-Fi module [41].
- Wemos D1 Mini—development board with ESP8266 microcontroller and Wi-Fi module [42].

**Figure 3:**
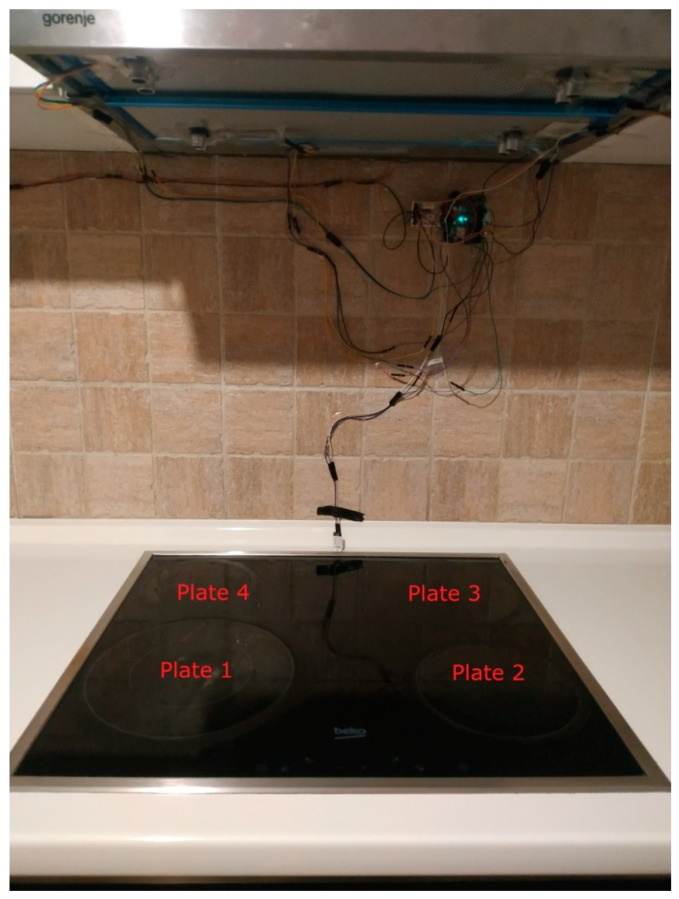
Experimental setup at hotplate location.

**Figure 4:**
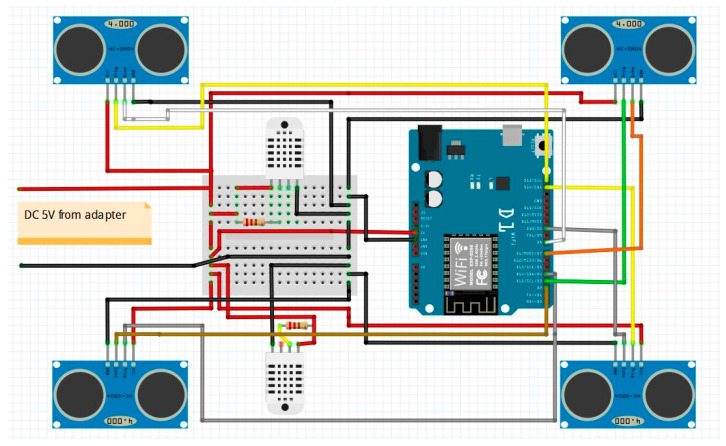
Schematic diagram of the proposed monitoring system.

The hardware prototype of the proposed experimental setup is shown in Figure 3, and a circuit diagram is shown in Figure 4. Four ultrasound sensors are attached to the aspirator hood and each of them is located above the corresponding hotplate plate. They are oriented orthogonally to the hotplate surface, to avoid mutual interference and receive accurate results of the measurements. Ultrasound sensors are connected to the digital pins of the Wemos D1 development board. 

One DHT sensor is located at the hotplate surface zone to monitor rapid temperature change, and the humidity sensor in this zone observes slower humidity change. Another DHT is placed on the wall, under the aspirator hood, near the ultrasound sensor. The Wemos D1 board is attached to the wall, in the same zone. The circuitry is powered by the USB power adapter. The Wemos D1 in the hotplate zone has a transmitter role and sends the data embedded in the GET request of the HTTP protocol.

On the remote side, there is a Wemos D1 Mini board connected to the USB port of the PC. Data sent from the hotplate location are received by this Wemos D1 Mini board. The board communicates with the PC via a serial port, where data are stored for further analysis. 

Based on the datasheet information, an ultrasound sensor HC-SR04 has a resolution of 3 mm, while the measuring distance range is from 2 to 400 cm [43]. Several factors, such as air temperature, humidity, pressure, and increased concentration of specific gases, may affect soundwave propagation velocity, and, consequently the measurement accuracy [44]. 

The speed of sound in the ideal gas at a temperature of 20 °C is approximately 343 m/s. Sound speed dependence of a temperature is given by the formula [45]:

*v* = 331.3 + k *T*, (1)

where k represents the constant and k = 0.607 m/s for every temperature change of 1 °C. *T* is the air temperature in °C. For the sake of better temperature compensation, we consider temperature measurements from both DHT sensors and calculate the arithmetic mean of those values. That becomes the input parameter *T* in Equation (1).

To calculate the distance based on the previously determined velocity *v*, the following formula was used:

*S* = *v* Δ*t*/2, (2)

where Δ*t* is the time interval between the soundwave transmission and the echo reception. 

In our experiments, we adopted a centimeter resolution, because finer distance variations are not considered to be important for the decision-making process. Thus, we used a round function in the program code to obtain integer values of distances measured by the ultrasound sensors. 

## 5. Results and Discussion

The goal of this section is to present the results of several tests performed to evaluate the performance of the proposed decision-making system and corresponding monitoring setup. All the scenarios discussed in Section 2.2 are considered and evaluated through the experiments. Special attention is given to the performance of the notification system that is essential for successful finalization of the cooking process. Test scenarios are designed to be as similar to the ordinary cooking process as possible. Results for both the monitoring and decision-making systems are presented and analyzed in detail. Results for real food preparation processes carried out by the older person are presented at the end.

Within the performed experiments, the following parameters where used:

- distance from the sensor to the plate is *D* = 57 cm;
- cookware utensil zone *X* = 10 cm, since smaller pots are used;
- alarm timers according to Section 3.1.3.

### 5.1. Simulation Results

**Test scenario 1:** *Plates are turned off and there are no objects placed*

**Figure 5:**
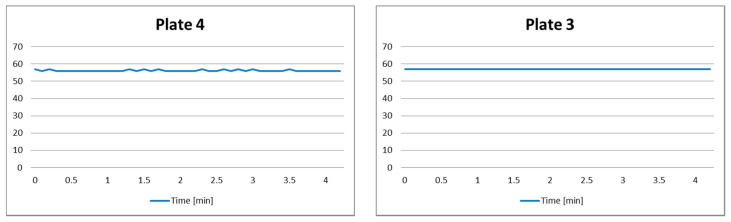

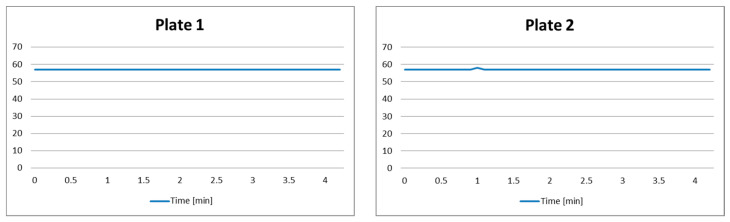
Test results of Scenario 1.

This scenario corresponds to Case 1.1, and is aimed to show that there is no interference among sensors when plates are turned off and empty. The obtained results are shown in Figure 5. We notice that there is almost constant distance *x*(*t*) = *D*. Small and random variations that occur are a consequence of the imperfect sensor installation and precision, as well as a rounding of the measured distances. 

The decision-making system will permanently remain in State Q1 regardless of small variations, and the system will proceed with monitoring. 

**Test scenario 2:** *Plates are turned off and there are some objects over it*

The aim of this scenario is to test the functionality of the implemented monitoring and decision-making system when all plates are turned off but there are some objects on them. 

*Setup*: Plate 1 had a pot on it for the whole time of experiment, while another pot is put on Plate 3 after 5 min.

*Expected system behavior*: The system notifies the user that the pot remains on the turned-off plate for a longer time than the predefined alarm threshold AT_1_ (which is set to 10 min).

**Figure 6:**
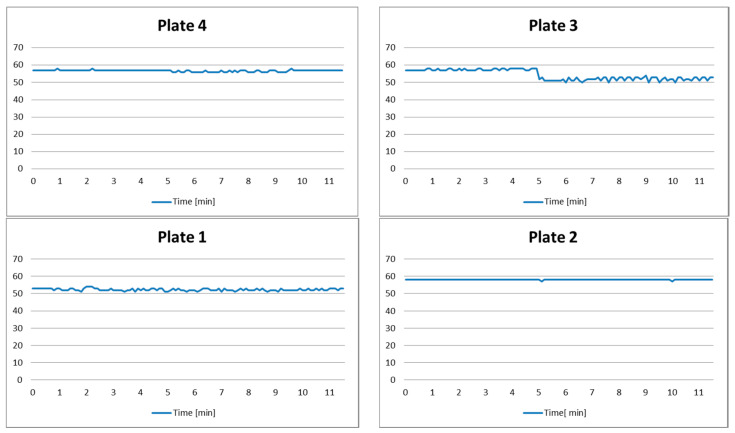
Test results of Scenario 2.

The measurement results are shown in Figure 6, and photo samples and decision-making systems inputs, states, and outputs are given in Table 5. One may notice that during the first 5 min of the experiment, the pot is present only on Plate 1. In that case, distance input is *d* = 10, while for the other plates it is *d* = 00. Consequently, a decision-making system makes the transition to State Q1 for Plate 1 and remains in State Q0 for the other plates. Input parameters remain the same until the 5th minute of the experiment, when a new pot is put on Plate 3. The decision-making system is in State Q1 for Plates 1 and 3, but remains in State Q0 for the other two plates. Since the pot remains on Plate 1 for a longer than 10 min, without user interaction, the alarm threshold AT1 is exceeded. The system state for Plate 1 is changed from Q1 to Q2 and a corresponding notification is triggered (output O_1_ for Plate 1).

**Test scenario 3:** *Plates 3 and 1 are turned on and there are no objects on them*

The goal of this test scenario is to check the measurement performance of the proposed system and to analyze if the decision-making system is capable of identifying Case 2.1 and generating the expected outputs and corresponding notifications. 

*Setup*: Plate 3 is turned on for a half of minute after the experiment starts, while Plate 1 is turned on after 2.5 min. The duration of the experiment is 4 min.

*Expected system behavior*: The system notifies the user that Plate 3 is turned on without the pot on it for a longer than predefined alarm threshold AT_2_ (which is set to 3 min).

**Figure 7:**
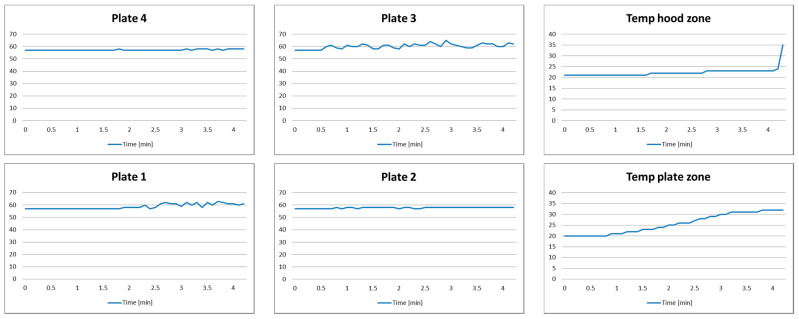
Test results of Scenario 3.

The measurement results for the distance and temperature are given in Figure 7. In the beginning of the experiment, all plates are turned off. One may notice that Plate 3 is turned on after half of a minute from the beginning of the experiment. After 2.5 min from the beginning of the experiment, Plate 1 is turned on. After turning on both Plate 3 and Plate 1, one can notice some smaller variations of measured distances for Plate 2 and Plate 4. This is a consequence of temperature variations. Specifically, the temperature at the plate zone increases much faster than temperature at the hood zone, which is normal for the relatively small duration of the experiment. Therefore, the temperature compensation for Plate 2 and Plate 4 was not performed ideally. 

As previously discussed, the impact of the temperature to the measurement results of the ultrasound sensor is significant. This is reflected as a reading of the distance value, which is higher than the maximum possible distance *D*. This might be useful for detecting which burner plate is turned on if this information is not available from the dedicated Hall sensor. 

Characteristic experiment photo samples and decision-making system inputs, states, and outputs for the Plate 3 are presented in Table 6. During the first half a minute, the system is in State Q1. After turning on the plate (*p* = 1), the system transitions to State Q3. Since it is identified that there is no pot on the plate, the system automatically passes to State Q4. The system remains within this state until the AT_2_ alarm threshold time is exceeded, when notification is generated (output O_2_). Since the user responded to the notification and turned off the plate, the emergency alarm is not triggered, and the cooking process is considered to have finished. This is reflected by the transition to State Q14.

Similar analysis could be performed for other plates. For Plate 1, the system transitions from State Q0 to Q3, and then to State Q4, but no alarms will be triggered upon finishing the experiment. For Plate 2 and Plate 4, the system remains within State Q0 during the experiment. 

**Test scenario 4:** *Regular cooking process including detection of boiling and evaporation*

This test scenario is designed to analyze the regular cooking process and to evaluate if the decision-making system is capable of detecting boiling and evaporation. 

*Setup*: Plate 2 is turned on and a pot with a lid is placed on it. At a certain moment, water in the pot will start to boil. After that, the user will interact with the cooking process. Finally, evaporation starts after several minutes.

*Expected system behavior*: The system detects the boiling process and the user interaction, as well as the trigger notification on evaporation process.

**Figure 8:**
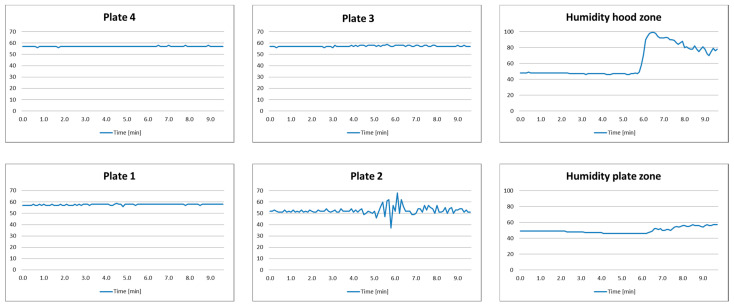
Test results of Scenario 4.

The measurement results are shown in Figure 8, while the characteristic experiment photo samples and decision-making system inputs, states, and outputs are given in Table 7. One can notice that during the first 5 min, an almost constant level (of about 51 cm) is detected. Since the plate is turned on and the pot is detected, this is recognized by the decision-making systems as an ongoing cooking process (State Q7). Small measurement variations are a consequence of temperature changes. After that, significant distance variations begin to occur. This is caused by the water boiling. Please note that humidity readings are slightly delayed, which is caused by the slow response time of the DHT22 sensor. The decision-making system will initiate transition to State Q8 to start the timer related to the possible boiling process. Since user interaction with the cooking process is detected (lifting the lid—measured distance 37 cm) there is no need to trigger the notification. This identified event (*d* = 11) will return the system to State Q7. Lifting the lid is identified by a significant increase of humidity. During the next 3 min, one can notice a constant decrease of humidity and a slight variation in the distance caused by an increased temperature and boiling process (the liquid produces waves that cause different reflections). This is identified as evaporation, and the system transitions to State Q8. Since there is no further user interaction with the cooking process, after 3 min (evaporation warning time WT_e_) the system transitions to State Q9 and generates a notification of possible evaporation to the user (output O_3_). After that, the user turned the plate off, so the emergency alarm was not triggered. 

### 5.2. Typical Cooking Process

A typical food preparation process with multiple plates and pots used is considered. The goal of this experiment is to analyze the decision-making system results for a real cooking process. 

**Figure 9:**
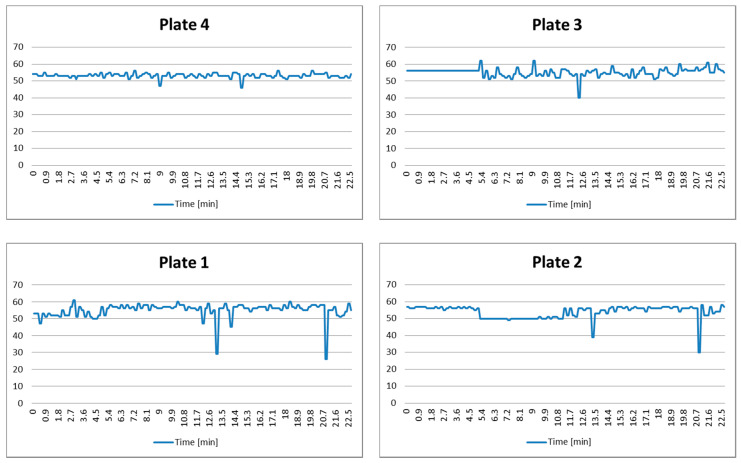
Measured distances for each plate during the typical cooking process.

The measured distances for each plate are shown in Figure 9. Characteristic photo samples of the performed cooking process and results of the corresponding decision-making for each plate are given in Table 8. During the first 4.5 min, Plate 1 is turned on and there is pot on it, while Plate 4 is turned off with a pot on it (distance value *x* = 53 is detected). Plates 2 and 3 are turned off and empty during this period (measured distance about 57 cm). Please note that small variations within readings for Plate 2 and Plate 4 are a consequence of temperature changes on the Plate 1. After that, the pot is removed from Plate 1 (distance higher than 57 cm due to increased temperature of the still-hot plate) and moved to Plate 3 that is turned on. Additionally, there is a pot detected on Plate 2 that remains turned off. Between 12.5 and 13 min from the beginning of the experiment, a user interaction with the cooking process is detected, first above Plate 1, and later above Plates 2 and 3. After that, the cooking process continues with the following statuses: Plate 1, Plate 2, and Plate 4 are turned off and empty, while Plate 3 is turned on with pot on it. Another interaction with the cooking process is detected at 20.7 min from the beginning of the experiment above Plate 1, when obviously some pot is placed on it. Movement is also detected above Plate 2 and the pot is not present on Plate 3 anymore (measured distances between 57 and 60 cm). At the end of the cooking process, there are pots detected on each plate.

Please note that there is only one notification to the user related to the pot placed on the turned-off plate. Since there were several user interactions with the cooking process, such a situation is expected. However, if we take into consideration all the plates together, even this notification is not necessary.

### 5.3. Discussion

In the previous subsections, experimental results are presented to clarify the capabilities and limitations of the proposed monitoring and decision-making system. One can notice that the proposed decision-making system is fully capable of identifying all possible scenarios discussed in Section 2, and could completely respond in accordance with the expected outputs. It is very important to emphasize that the user can be guided toward a successful finalization of the cooking process following instructions from the proposed decision-making system. Regarding the monitoring system, we found that overall experimental results fully correspond to the expected behavior of the system in all test scenarios.

To further evaluate the performance of the proposed monitoring and decision-making system, we performed a comparison with similar studies and summarized the results in Table 9. The study reported in [18] is based on a thermal camera. In [28], the authors used ultrasound and temperature sensors similar to those in our study, but with different positions. It can be observed that all systems can identify plate status and presence of the pot. Decision systems for these solutions enable triggering alarms for possible dangerous situations that are quite similarly identified in these studies. User interaction with the cooking process is considered in [18] in a quite similar manner to our system, although boiling and evaporation are not considered at all. In [28], the detection of boiling and evaporation could be identified, while the detection of user interaction with the cooking process in not possible due to the sensor positions. Finally, neither studies observed if the cooking process was successfully finished. Additionally, they did not provide notifications that could help the user to successfully finish the cooking process.

Comparing installation requirements, each system has some specific requirements. The least suitable is the system proposed in [28], since it requires quite a large surface on the working area to be able to monitor each plate. This also means that it may disturb a regular cooking process, especially bearing in mind that the target group are older people. An additional important aspect is the visibility of the monitoring system. Specifically, as mentioned before, most older adults do not like to be constantly monitored, and therefore systems should be as unintrusive as possible. A system based on thermal camera is the most expensive. 

From the above analysis, one can conclude that the proposed system outperforms the other two related studies in almost all aspects. It can identify more scenarios in the kitchen environment. Furthermore, the proposed decision-making system provides notifications that could lead the user through the coking process. Moreover, it can recognize if the cooking process is successfully finished. Finally, installation cost, pre-requirements, and visibility by the user are more advanced compared to other analyzed solutions.

However, there are some issues that could be improved. For instance, in our experiments, each plate has been monitored and analyzed separately. It would be interesting to test the behavior of the system and check its robustness with the joint plate analysis. Additionally, the position of the temperature and humidity sensors could be reinvestigated. It could be evaluated if their placement above each plate or next to each plate would improve temperature compensation. Furthermore, more accurate ultrasound sensors could improve results regarding the detection of boiling and evaporation processes, while in other cases, enhanced precision will not provide additional benefits. 

In the current form, the proposed monitoring system could be implemented only in kitchens equipped with a hood or kitchen elements above the cooker. Otherwise, some modification of the kitchen setup or proposed system should be required. Another limitation of the proposed system is that it is designed and tested only for electrical flat-board cookers.

## 6. Conclusions

The monitoring and decision-making system proposed in this paper enables identification of activities of senior people in a kitchen. The proposed monitoring system consists of four ultrasound sensors which detect the presence of pots on the plates, as well as user activities over the cooking surface. Each of these sensors is responsible for its plate, but there is also the possibility to detect some conditions around that plate. Experimental results showed that information from these sensors is not sufficient for some situations. Therefore, we added two temperature and humidity sensors to the monitoring system. One is responsible for measuring temperature and humidity near the hotplate surface, and the other near the ultrasound sensors, which are mounted on the hub.

Additionally, we proposed an original decision-making system, capable of detecting anomalous and possibly critical situations. If the system detects conditions that indicate that the user has forgotten about the cooking process, an appropriate warning is sent to the user. Furthermore, if the condition is recognized as potentially dangerous, an alarm is sent to the user and to (in)formal caregivers. Additionally, some precautionary measures can be taken if the cooker and kitchen appliances have the necessary functionalities. 

One of the key features that the proposed monitoring and decision-making system enables is the ability to lead a user to successfully finish the cooking process. Most of the previous studies have focused just on the identification and reaction to possibly critical and dangerous situations. The contribution of this paper is the introduction of a novel decision-making system, which includes an advanced notification system. The goal of this system is to notify the user about the possible failure of the cooking process. It is also capable of leading the user towards a successful finalization of the cooking process. Having in mind that food preparation, including cooking, might be quite challenging for older people, especially those with cognitive disabilities, this feature has the potential to improve user satisfaction and wellbeing in general. The proposed notification and decision-making system could be easily integrated in some already-developed daily activity monitoring systems that are available on the market.

The decision-making system can also provide information that could be used to derive lifestyle patterns and the daily functioning of the user. Based on this information, medical and social services, as well as relatives, can take appropriate action to improve the quality of life and health protection of the elderly person. Future research will be primarily focused on previously identified issues regarding improvements to the proposed system to enhance performance and overcome limitations. Additional experiments about the position of the sensors, using more accurate devices, and joint plate-state analysis will be performed. Additionally, the possibility to expand the application of the proposed system will be addressed. Using this system in different environments will provide new information about required functionalities, leading to the identification of further research directions. Most contributions are expected within the reasoning module and the lifestyle pattern recognition, which could lead to the design of an extensive and useful daily functioning reporting system. Additionally, the proposed monitoring system will be tested by other decision-making systems proposed in the literature to check its robustness and applicability to other known systems. An additional very important improvement could be the integration of derived user patterns and past decisions to enhance the stability and confidentiality of the system. Information from previous decisions could also lead to a more personalized system. 

## Figures and Tables

**Table 1:** Values of the distance parameter *d*.

Input Value	Condition	Description
*d* = 00	x(t)=D	no object is detected on the plate
*d* = 01	x(t)>D	no object is detected on the plate (but temperature is high) *
*d* = 10	D−X<x(t)<D	pot is detected on the plate
*d* = 11	x(t)<D−X	interaction with cooking process, such as hand movement, lid movement or similar is detected

* Distance measured by ultrasound sensor is higher than maximal distance *D*. This occurs due to the increased temperature caused by the hot plate. More details are provided in Section 5.

**Table 2:** Values of the alarm parameter *a*.

Input Value	Condition	Precondition	Description
*a* = 00	$t>AT_{1}$	p(t)=0, d(t)=10	Case 2.1—plate is turned off and object is detected on it longer than period *AT*_1_
*a* = 01	$t>AT_{2}$	p(t)=1, d(t)=01	Cases 3.1 and 3.2—plate is turned on, no object is detected on it longer than period *AT*_2_
*a* = 10	$t>AT_{3}$	p(t)=1, d(t)=10	Case 4.4.—no interaction with cooking process longer than period *AT*_3_
*a* = 11	$t_{1}>AT_{c}$		No reaction to possibly critical event longer than period *AT_c_*

**Table 3:** Values of the warning parameter *w*.

Input Value	Condition	Precondition	Description
*w* = 00	$t_{w}<WT_{b}$	p(t)=1 x(t−1)=x(t) h(t−1)≈h(t)	no boiling conditions
*w* = 01	$t_{w}>WT_{b}$	p(t)=1 x(t−1)=x(t) h(t−1)<h(t) or p(t)=1 x(t−1)>x(t) h(t−1)<h(t)	constant distance, humidity increased (case with pot) distance decreased and humidity increased (case without lid)
*w* = 10	$t_{w}<WT_{e}$	p(t)=1, x(t−1)=x(t) h(t−1)≈h(t)	no evaporation conditions
*w* = 11	$t_{w}>WT_{E}$	p(t)=1 x(t−1)=x(t) h(t−1)>h(t) or p(t)=1 x(t−1)<x(t) h(t−1)>h(t)	constant distance, humidity decreased (case with pot) distance increased, humidity decreased (case without lid)

**Table 4:** Decision-making systems implemented as Moore finite-state machine.

State	Output	Inputs				Next State
		*p*	*d*	*w*	*a*	
Q0	O_0_	0	-	-	-	Q0
		0	10	-	-	Q1
		1	-	-	-	Q3
Q1	O_6_	0	10	-	00	Q2
		0	11	-	-	Q0
		1	10	-	-	Q7
Q2	O_1_	-	-	-	-	Q0
Q3	O_6_	1	01	-	-	Q4
		1	10	-	-	Q7
		0	-	-	-	Q14
Q4	O_6_	0	-	-	-	-
		1	01	-	01	Q5
		1	10	-	-	Q7
Q5	O_2_	-	-	-	-	Q6
Q6	O_5_	0	-	-	-	Q14
		1	01	-	01	Q5
		1	10	-	-	Q7
Q7	O_6_	1	10	01 or 11	-	Q8
		1	10	-	10	Q11
		1	01	-	-	Q3
		0	-	-	-	Q14
Q8	O_6_	1	10	01 or 11	11	Q9
		1	11	-	-	Q7
		1	01	-	-	Q3
Q9	O_3_	-	-	-	-	Q10
Q10	O_5_	0	-	-	-	Q14
		1	11	-	-	Q7
		1	10	-	11	Q9
		1	01	-	-	Q3
Q11	O_6_	1	10	-	11	Q12
		1	11	-	-	Q7
		0	-	-	-	Q14
Q12	O_4_	-	-	-	-	Q13
Q13	O_5_	0	-	-	-	Q14
		1	11	-	-	Q7
		1	10	-	10	Q12
		1	01	-	-	Q3
Q14	O_7_	0	00	-	-	Q0

**Table 5:** Analysis of the results for test scenario 2: Plate 1 and Plate 3.

photo	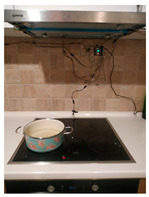	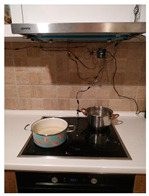	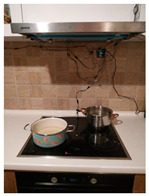
Time [min]	*t* = 0	*t* = 5	*t* = 10.1
Plate status	0	0	0
distance	Plate 1—10 Plate 3—00	Plate 1—10 Plate 3—00	Plate 1—10 Plate 3—00
alarm	-	-	00
warning	-	-	-
State	Plate 1—Q1 Plate 3—Q0	Plate 1—Q1 Plate 3—Q1	Plate 1—Q2 Plate 3—Q1
Output	Plate 1—O_0_ Plate 3—O_0_	Plate 1—O_0_ Plate 3—O_0_	Plate 1—O_1_ Plate 3—O_0_

**Table 6:** Analysis of the results for test Scenario 3: Plate 3.

photo	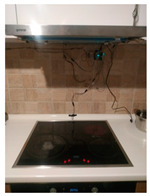	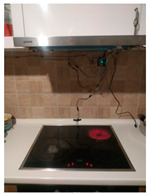	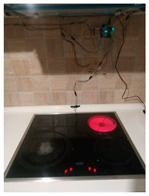	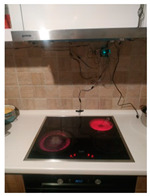	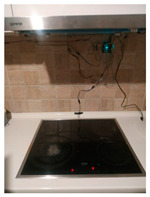
Time [min]	*t* = 0	*t* = 0.5	*t* = 2.5	*t* = 3.5	*t* = 4
*x*—Plate 3	57	57	64	63	61
Plate 3 status	0	1	1	1	0
distance	00	01	01	01	01
alarm	-	-	-	01	-
warning	-	-	-	-	-
State	Q0	Q3 -> Q4	Q4	Q4 -> Q5	Q14
Output	O_0_	O_6_	O_6_	O_2_	O_7_

**Table 7:** Analysis of the results for test Scenario 4: Plate 2.

photo	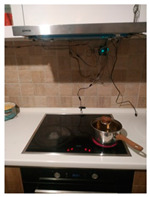	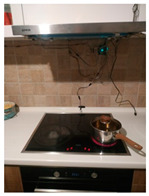	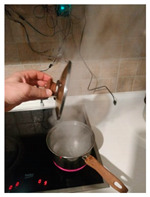	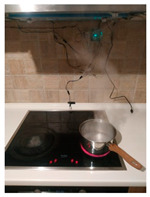	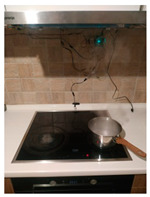
Time [min]	*t* = 0.5	*t* = 5	*t* = 5.1	*t* = 9	*t* = 9.3
*x*	51	60	37	55	61
*h*	48	71	99	75	73
Plate status	1	1	1	1	0
distance	10	01	11	10	01
alarm	-	-	-	-	-
warning	-	-	-	11	-
State	Q7	Q7 -> Q8	Q8 -> Q7	Q8 -> Q9	Q14
Output	O_6_	O_6_	O_6_	O_4_	O_7_

**Table 8:** Analysis of the results for typical cooking process.

photo	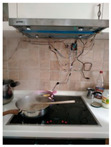	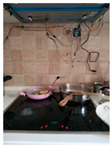	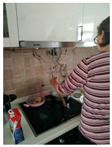	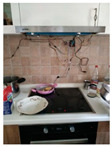	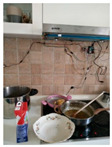
Time [min]	*t* = 0-4	*t* = 5–12	*t* = 12.7–13	*t* = 21	*t* = 21.5
*x*	Plate 1—49–55 Plate 2—57 Plate 3—57 Plate 4—53	Plate 1—58 Plate 2—51 Plate 3—50–60 Plate 4—50–60	Plate 1—28 Plate 2—40 Plate 3—40 Plate 4—40	Plate 1—50–52 Plate 2—30 Plate 3—51 Plate 4—55	Plate 1—50–52 Plate 2—50–52 Plate 3—50–52 Plate 4—55
*p*	Plate 1—1 Plate 2—0 Plate 3—0 Plate 4—0	Plate 1—0 Plate 2—0 Plate 3—1 Plate 4—0	Plate 1—0 Plate 2—0 Plate 3—1 Plate 4—0	Plate 1—0 Plate 2—0 Plate 3—1 Plate 4—0	Plate 1—0 Plate 2—0 Plate 3—0 Plate 4—0
States	Plate 1—Q7 Plate 2—Q0 Plate 3—Q0 Plate 4—Q2	Plate 1—Q14 Plate 2—Q2 Plate 3—Q7 Plate 4—Q2	Plate 1—Q14 Plate 2—Q2 Plate 3—Q7 Plate 4—Q2	Plate 1—Q14 Plate 2—Q0 Plate 3—Q7 Plate 4—Q0	Plate 1—Q14 Plate 2—Q0 Plate 3—Q14 Plate 4—Q0
Output			Plate 2—O_2_ Plate 4—O_2_		

**Table 9:** Comparison with other solutions.

	Ref [18]	Ref [28]	Proposed System
Empty and turned-off plate	Yes	Yes	Yes
Empty and turned-on plate	Yes	Yes	Yes
Turned-off plate with pot on it	Yes	Yes	Yes
Turned-on plate with pot on it (regular cooking process)	Yes	Yes	Yes
Boiling detection	No	Yes	Yes
Evaporation detection	No	Yes	Yes
User interaction with cooking process (stirring pot, removing lid, using mixing spoon)	Yes	No	Yes
Identifying successfully finished cooking process	No	No	Yes
Alarming system—possibly critical situations	Yes	Yes	Yes
Notification system—corrective actions to safely complete cooking process	No	No	Yes
Installation costs	High (>500 EUR)	Low (<100 EUR)	Low (<100 EUR)
Installation pre-requirements	No	Yes (sufficient space from each side of hotplate to place sensors)	Yes (existence of hood or upper kitchen elements)
Visible by the user	High	High	Low
Cooking process obstruction	Moderate	Moderate/high	Low
